# Serine Octamer Substitution Reactions With α‐Hydroxy Acids

**DOI:** 10.1002/rcm.70133

**Published:** 2026-07-02

**Authors:** Keerthana Unni, Brison A. Shira, Dylan T. Holden, Thalappil Pradeep, R. Graham Cooks

**Affiliations:** ^1^ Department of Chemistry Purdue University West Lafayette Indiana USA; ^2^ DST Unit of Nanoscience (DST UNS) and Thematic Unit of Excellence (TUE), Department of Chemistry Indian Institute of Technology Madras Chennai India

## Abstract

**Rationale:**

The amino acid serine forms chiral magic number clusters, which can be studied using mass spectrometry (MS). Serine octamer clusters are also known to incorporate one or two nonserine monomers with serine monomers to make up the octamer.

**Methods:**

A Thermo LTQ ion trap mass spectrometer with nanoelectrospray ionization (nESI) was used to study cluster formation by both kinetically and energy‐resolved methods. This study validates past observations on the homochiral preference of serine octamer during its association with other nonserine monomers.

**Results:**

A substrate scope covering α‐hydroxy acids and related molecules was studied. The chiral preference was investigated with tandem MS and the importance of salt bridge formation and α/β‐carbon hydrogen bonding was recognized.

**Conclusions:**

Weak interactions involving steric forces, salt bridges, ion–dipole interactions, and hydrogen bonds control stereoselective and chiroselective properties of the serine octamer cluster.

## 1 Introduction

Noncovalent interactions are encountered throughout physics and chemistry and are especially important in biological systems [1, 2]. Weak intermolecular forces establish structural organization in biomacromolecules, including proteins and nucleic acid [3, 4]. Cases in which simple molecular systems exhibit properties such as strong chiral associations based on noncovalent interaction are therefore fundamentally interesting [5, 6], as are the methods of studying them [7, 8, 9, 10, 11, 12].

**FIGURE 1:**
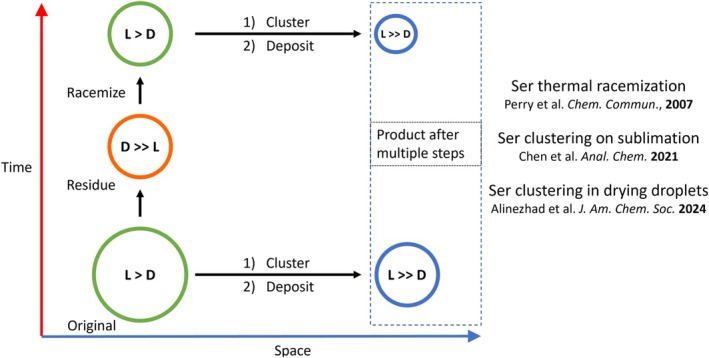
Molecular clustering plus racemization increases enantiomeric excess (e.e.) without chemical change. Given a small starting e.e. (shown as L > D), clustering and then deposition provides two populations (blue and orange), each enriched in one enantiomer (L > > D and D > > L). If one of these populations is subjected to a racemizing event in its new location, it would return to the original e.e. condition, which would allow the chiroselective process to repeat. References demonstrating relevant aspects are provided in the figure.

One instance is the serine octamer magic number cluster, which was studied as a protonated molecule in our experiments. Serine monomers cluster chiroselectively when they are generated from microdroplet sprays [13, 14, 15, 16, 17, 18]. This cluster's homochiral behavior results from its highly symmetrical D_4_ structure, which consists of two 4‐monomer layers linked by NH_3_^+^/COO^−^ salt bridges and hydrogen bonds connecting the two layers [13, 19]. The octamer imposes selectivity during substitution when one or two serine molecules are replaced by other biomolecules that possess moieties capable of participating in the noncovalent network [20, 21, 22]. It is known that the serine octamer chiroselectively substitutes amino acids and glucose, incorporates H_3_PO_4_, and forms complexes with transition metals [23, 24, 25, 26, 27]. Chirally selective substitution into nanoscale clusters is interesting because it is unassisted by outside chiral forces [18, 28, 29], so given any degree of inherent asymmetry in the monomer population, in principle, it can lead to chiral enrichment, even to homochirality, provided only that the selection is followed by segregation of the substituted cluster from the monomer [30, 31]. This situation is illustrated schematically in Figure 1.

In the present study, the substitution reaction was studied by acquiring data on clusters containing both serine and another monomer. Our aim is to probe octamer substitution by examining the cluster's behavior with molecules of lower proton affinity (PA) than amino acids. The interactions of amino acids with the charged cluster are driven by the salt bridge binding of basic groups at the charge site. Hydroxy acids, which have lower PA than amino acids, are expected to be more sensitive to other determinants of substitution, namely, sterics and the arrangement of hydrogen bonding groups about the chiral center [32].

Hydroxy acids have featured in studies of stereochemistry since Pasteur's early work with tartaric acid (TA) [33, 34]. Moreover, hydroxy acids are possibly relevant to life's chemical evolution, based on findings from simulated prebiotic chemistry [35, 36, 37, 38, 39]. We examined various hydroxy acids to identify the properties necessary for stereoselective and chiroselective incorporation into the serine‐based octamer. Table 1 presents the α‐hydroxy acids and related molecules studied and the results obtained.

**TABLE 1:** Substrates subjected to clustering interactions with serine.

Sl. no.	Substrate	Octamer substitution?	Chiroselective?
1	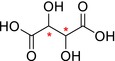 Tartaric acid (TA)	✔	✔
2	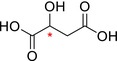 Malic acid (MA)	✔	✔
3	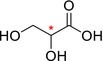 Glyceric acid	✔	✔
4	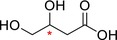 3,4‐Dihydroxybutyric acid	Weak	N/A
5	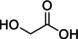 Glycolic acid	✘	✘
6	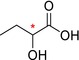 2‐hydroxybutyric acid	✘	✘
7	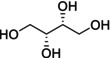 Threitol	Weak	N/A

By studying the substrate scope of molecules with hydroxyl and carboxylic acid functions and two to five backbone carbons, we have endeavored to identify the characteristics that allow serine octamer association (viz., substitution). Using nanoelectrospray ionization (nESI) with pulled borosilicate capillary emitters, we conducted MS analysis using a quadrupole ion trap instrument (Thermo LTQ). Our general finding is that molecules, which can participate in the salt bridge structure, cluster efficiently with serine, with additional advantage conferred by α and β hydroxyl groups (but not β and γ). Molecules with too few carbons do not fit into the vacancy created by removing a serine molecule from the octamer, whereas much larger molecules cannot be accommodated. Molecules of intermediate size are incorporated provided they appropriately participate in the cluster's noncovalent interaction network. In those cases where clustering occurred, we investigated the chiral preference of mixed serine octamer clusters.

## 2 Results and Discussion

**FIGURE 2:**
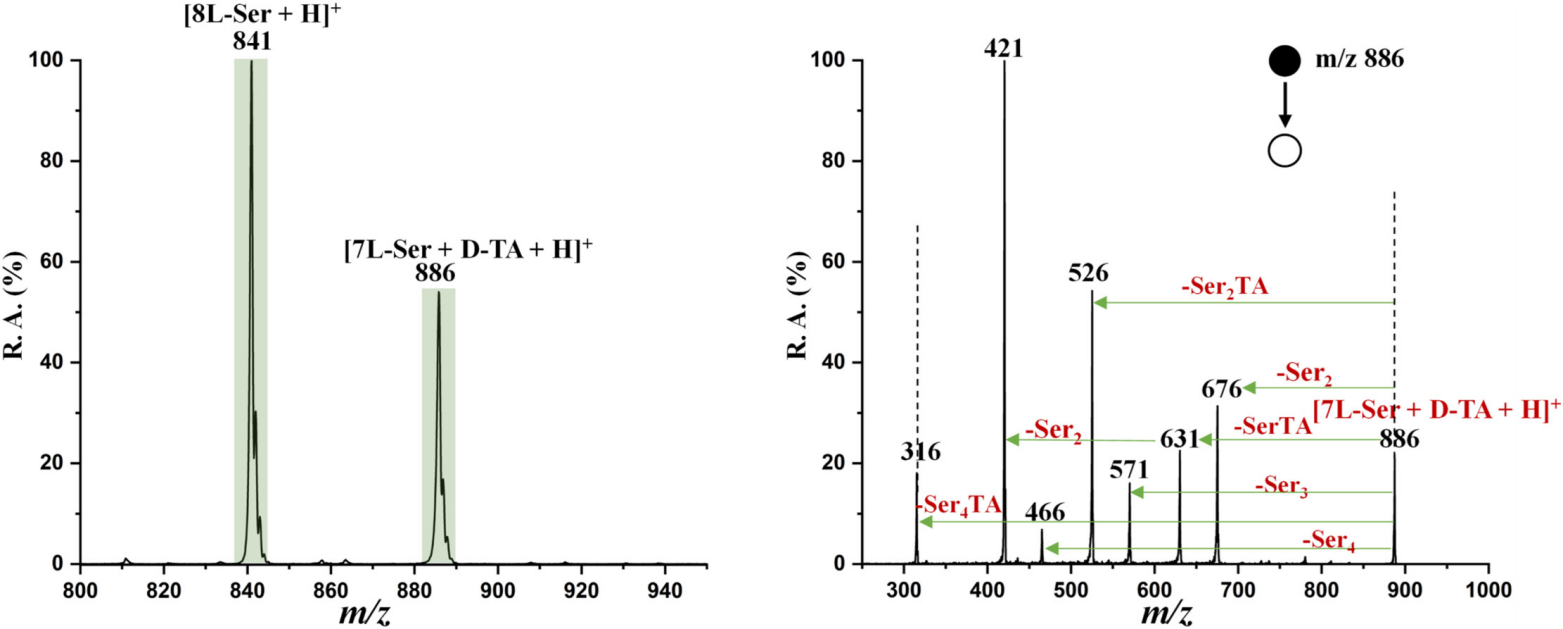
(a) nESI mass spectrum of aqueous L‐serine (8 mM) and D‐tartaric acid (2 mM), showing singly substituted serine octamer, (b) tandem mass spectrum showing the CID of [7L‐Ser + D‐TA + H]^+^ at *m/z* 886.

Experiments began by examining how serine interacts with the stereoisomers of TA. Figure 2a shows the region of the positive‐ion mass spectrum obtained using nESI for an aqueous solution of L‐serine and D‐TA dissolved in water (also, Figure S1 shows the octamer [7L‐Ser + D‐TA + H]^+^ in the range of *m/z* 500–1000). The singly substituted octamer, [7L‐Ser + D‐TA + H]^+^, where TA is tartaric acid, appears with good intensity at *m/z* 886. Tandem MS (MS/MS) analysis using collision‐induced dissociation (CID) confirmed this assignment (Figure 2b), with fragment ions corresponding to the loss of di‐, tri‐, and tetrameric serine clusters, along with similar clusters containing TA. Prior to the serine substitution experiments, the TA clusters were analyzed by mass spectrometry (Figure S2).

**FIGURE 3:**
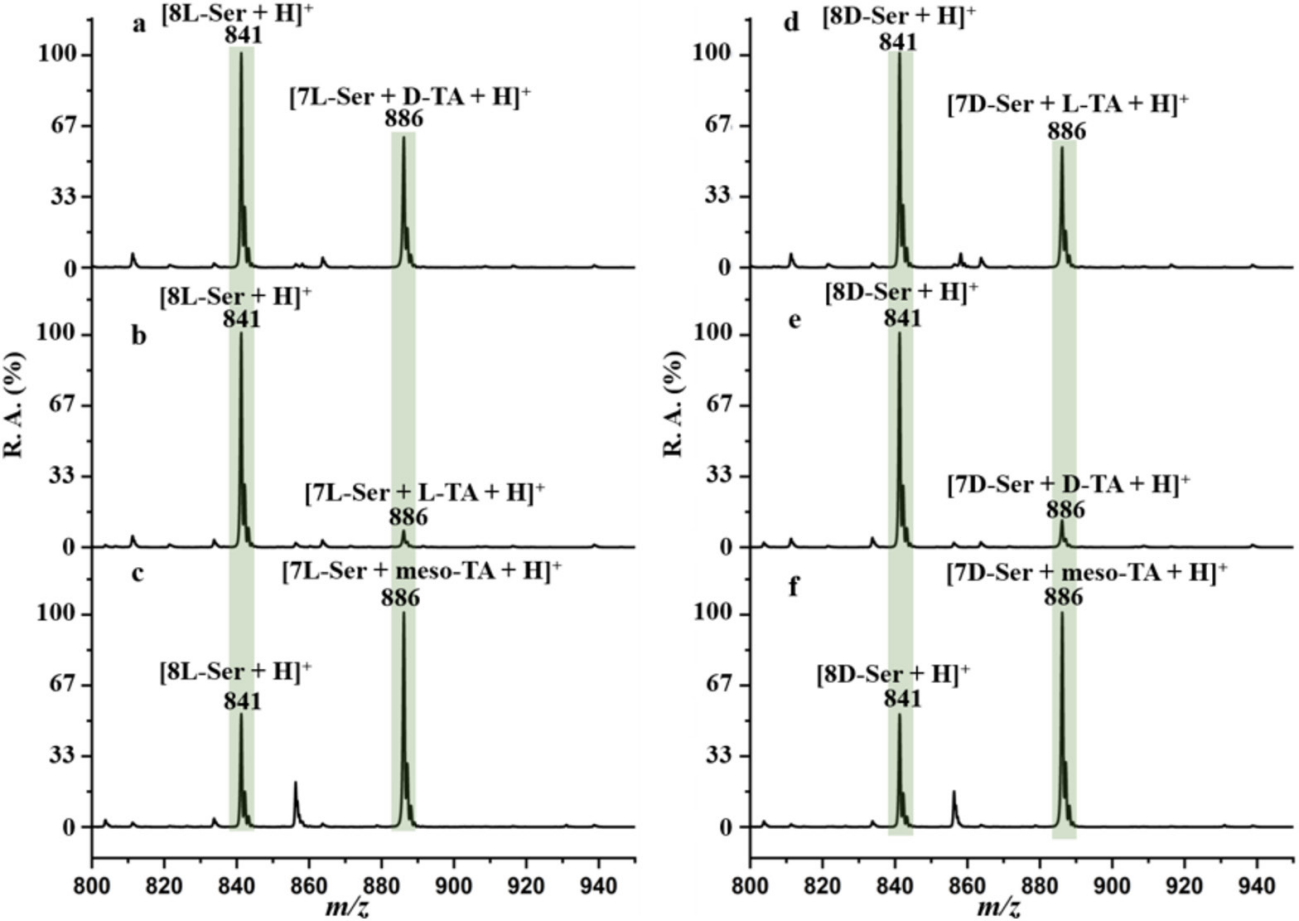
Mass spectra showing substitution as a function of stereochemistry. (a) L‐serine and D‐TA, (b) L‐serine and L‐TA, (c) L‐serine and meso‐TA, (d) D‐serine and L‐TA, (e) D‐serine and D‐TA, and (f) D‐serine and meso‐TA.

Chiroselectivity was observed with the D‐enantiomer of TA preferring substitution into the L‐serine octamer (Figure 3). Figure 3a–c displays the chiroselective patterns of L‐serine with D‐, L‐, and meso‐TA. Validating the data quality, Figure 3d–f shows the preference in reverse, with D‐serine pairing with L‐TA. The achiral meso‐TA stereoisomer shows the most abundant clustering interaction. A differentiating feature between the L‐/D‐TA and meso‐TA is its mirror plane reflection symmetry (*σ*) between the α and β carbons. Added symmetry may facilitate incorporation in the cluster because both hydroxyls can point toward the other polar moieties of the cluster while keeping the two carboxylic acids in a symmetrical position, preserving the cluster's ordered structure and noncovalent contacts. Such a conformation is not available to the D‐ and L‐enantiomers.

Measuring the stereoselectivity and enantioselectivity of the clustering process as shown in Figure 3 is a kinetic method of analysis [40]: Constant proportions of monomers are sprayed, and they experience the same reaction time and conditions. The relative abundance of a given product as a function of stereochemistry indicates the chiral preference. Alternatively, the strength of the noncovalent forces that hold the cluster together can be measured by energy resolved CID. In this implementation, a given ion is isolated and subjected to variable CID energies. When fragment ion abundances are plotted with respect to the CID energy, the breakdown curves indicate how much energy is required to drive the dissociation. We note that this general approach has been used to obtain relative thermochemical information [41]. When this approach is applied to the much simpler proton‐bound dimer, atomic or molecular cation, or even an electron, quantitative relative free energies may be obtained [42].

**FIGURE 4:**
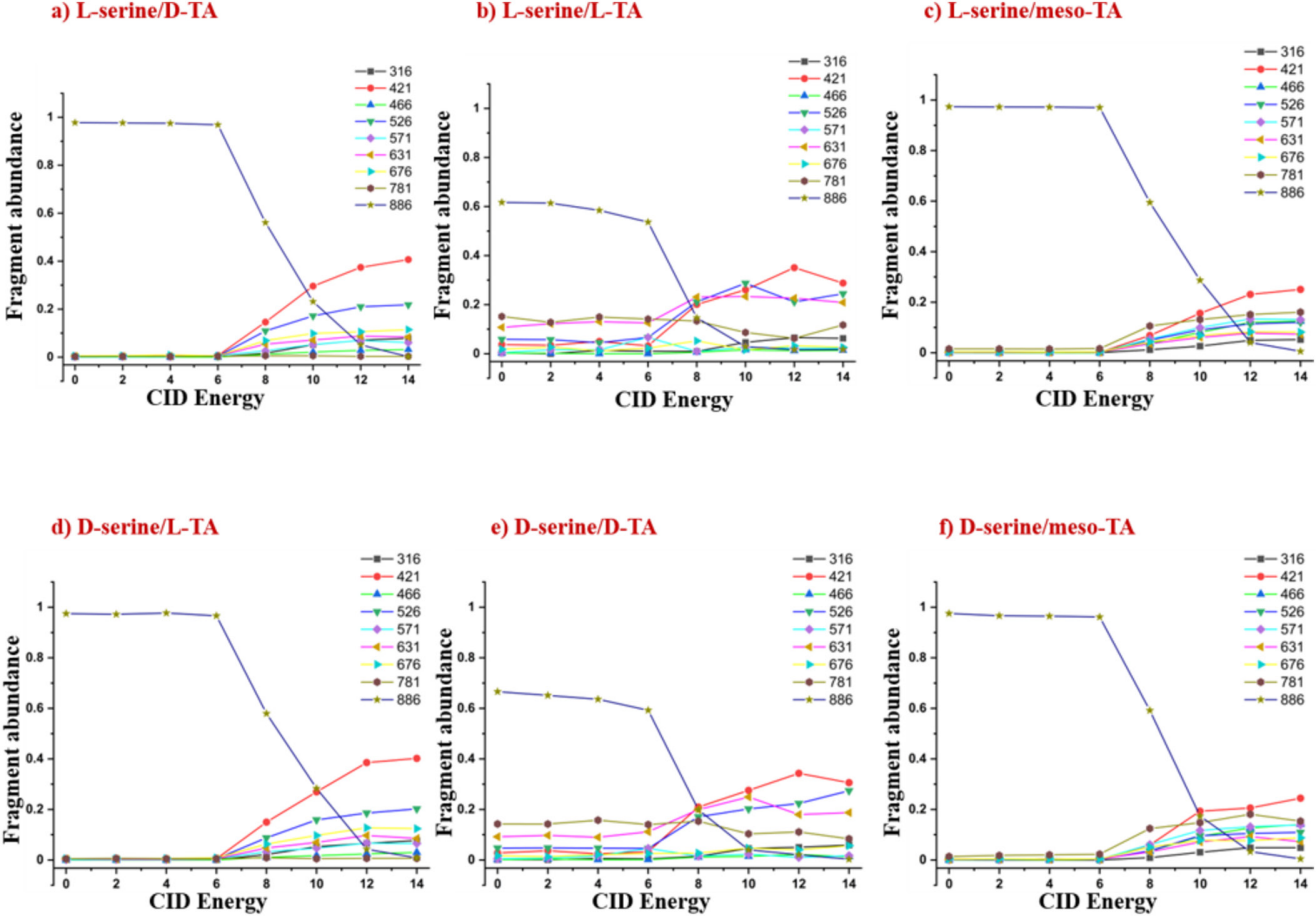
Breakdown curves of [7Ser + TA + H]^+^. CID energy is manufacturer's unit. (a) L‐serine/D‐TA, (b) L‐serine/L‐TA, (c) L‐serine/meso‐TA, (d) D‐serine/L‐TA, (e) D‐serine/D‐TA, and (f) D‐serine/meso‐TA.

To better understand the forces causing octamer formation, we examined how fragmentation varies with CID energy for the [7Ser + TA + H]^+^ ions (Figure 4). The collision energy values shown in the breakdown curves correspond to the instrument's CID energy settings and are reported in the manufacturer's unit. The curves were plotted as fractional ion abundances. Comparison of the breakdown curves (Figure 4a–c) shows that L‐Serine‐, D‐TA‐, and meso‐TA‐containing clusters remain more resistant to fragmentation, with major precursor depletion beginning at approximately eight CID energy. In contrast, the L‐TA‐containing cluster shows fragmentation at lower CID energy, indicating that this mixed octamer is less stable under collisional activation. Validating the data quality, Figure 4d–f shows breakdown curves for D‐Serine.

According to the literature, the breakdown curve of the protonated L‐serine octamer indicates that dimer loss is the dominant and lowest energy dissociation pathway, whereas the loss of an odd‐numbered monomer is unfavorable. Subsequent mass selection, dissociation, and mass analysis (MS^3^) of the major dissociation product, the protonated hexamer, also demonstrate the loss of a serine dimer to yield the protonated tetramer.

In contrast, the TA‐substituted serine octamers exhibit distinctive fragmentation pathways. The L‐serine octamer substituted with D‐TA predominantly loses a serine dimer as well as a mixed Ser–TA dimer (Figure S3). However, the octamers substituted with L‐TA and meso‐TA show an additional fragment at *m/z* 781, corresponding to the neutral loss of a serine monomer. These observations suggest that incorporating D‐TA preserves the characteristic dimer‐loss dissociation behavior of the native serine octamer, whereas substitution with L‐TA or meso‐TA alters the fragmentation pathway, leading to monomer loss and indicating differences in stability and binding interactions within the cluster. A similar MS/MS pattern was observed for D‐serine also (Figure S4). Taken together, the abundance measurements and CID breakdown curves indicate that the stability and fragmentation of the mixed serine/TA octamers are strongly controlled by TA stereochemistry. The different dissociation behavior is most likely due to changes in the hydrogen‐bonding network of the octamer after substitution of one serine molecule by TA.

**FIGURE 5:**
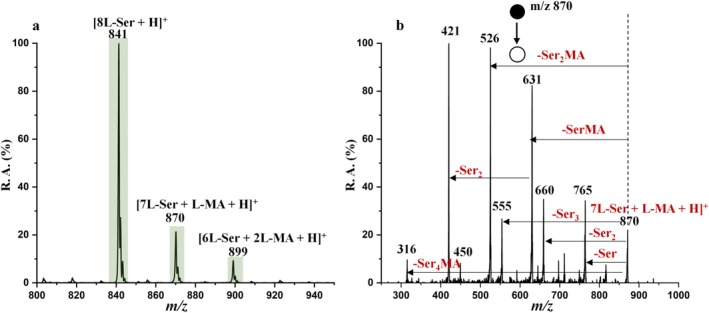
(a) nESI mass spectrum of L‐serine (8 mM) and L‐MA (2 mM) showing the formation of single and double substituted octamers, (b) MS/MS of [7L‐Ser + L‐MA + H]^+^.

Malic acid (MA) also exhibited stereoselective associations with a slight preference for pairing L‐serine with L‐MA over D‐MA. Figure 5a shows the nESI mass spectrum of L‐serine/L‐MA. The peaks at *m/z* 870 and 899 correspond to single and double MA substitution, [7Ser + MA + H]^+^ and [6Ser + 2MA + H]^+^, respectively. Figure 5b shows the MS/MS spectrum of [7Ser + MA + H]^+^, which includes the loss of neutral monomer, di‐, tri‐, and tetramers of serine, along with fragments containing both MA and serine. (Additional characterization is discussed in Figure S5).

**FIGURE 6:**
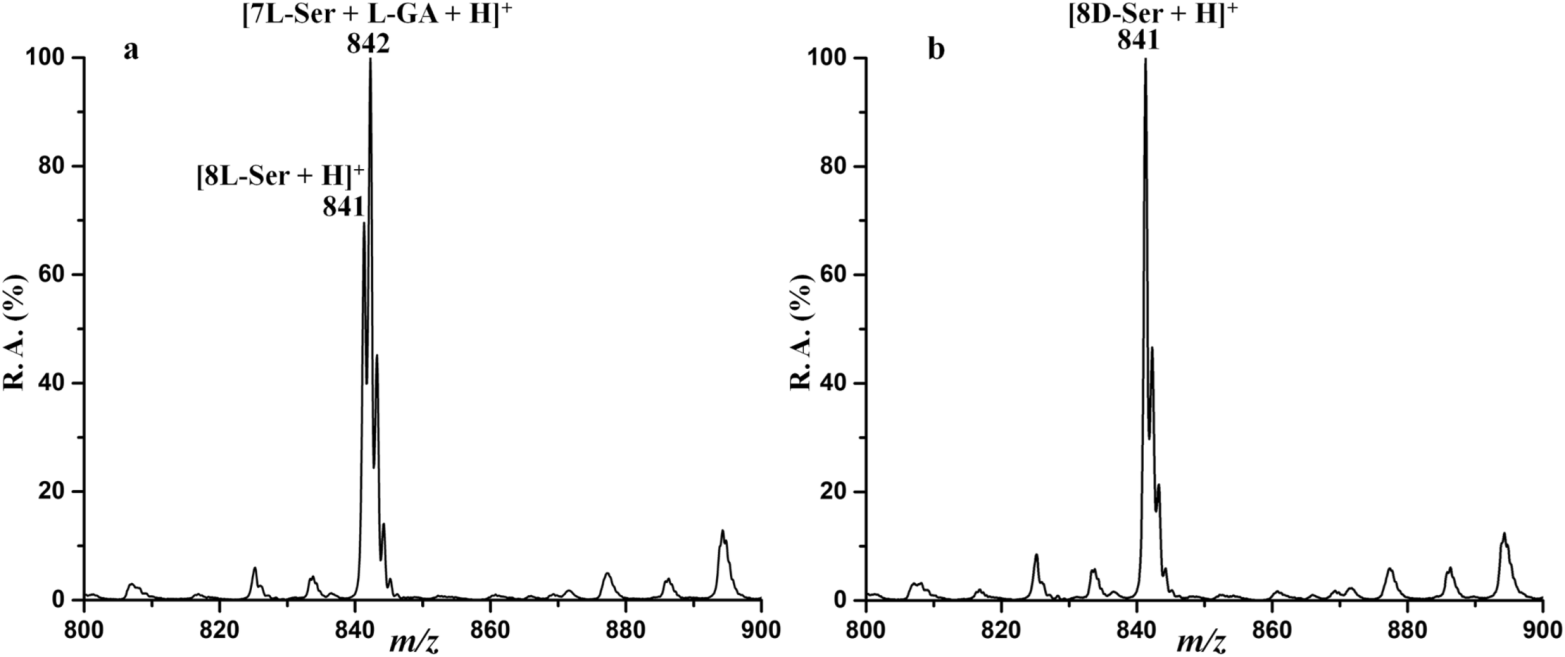
nESI mass spectrum of a mixture of (a) L‐serine (8 mM) and L‐glyceric acid (2 mM) showing the formation of the substituted product, (b) D‐serine (8 mM) and L‐glyceric acid (2 mM), indicating substitution is less favorable.

In addition to TA and MA, glyceric acid (GA) showed strong chiral selectivity, with [7L‐Ser + L‐GA + H]^+^at *m/z* 842, whereas the D‐serine/L‐GA cluster was not detected (Figure 6). To further confirm this, we performed substitution studies using different chiralities of serine and GA sodium salt in the presence of acid (Figure S6). L‐serine/L‐GA sodium salt and D‐serine/D‐GA sodium salt showed chiroselective substitution with a peak at *m/z* 842, whereas L‐serine/D‐GA and D‐serine/L‐GA did not exhibit any substitution. GA has a very similar structure to serine, with a hydroxyl instead of serine's amine. Even when an amine is unavailable to participate in a salt bridge, because GA closely matches the structure of serine, it does not significantly disrupt the larger structure and chiral preference is retained.

The observed differences in substitution efficiency and chiroselectivity among the hydroxy acids appear to arise from structural features that govern their accommodation within the Ser_8_ assembly. To investigate this relationship, a series of hydroxy acids and related polyols were examined.

Neither serine/glycolic acid (5) (Figure S7) nor serine/L‐2‐hydroxybutyric acid (6) (Figure S8) showed detectable substitution. This observation suggests that the presence of a single hydroxyl group and carboxyl group is insufficient to promote incorporation into the octamer. In contrast, partial replacement of the carboxylic acid functionalities of TA by hydroxyl groups, as in threitol (7) (Figure S9), resulted in only very weak substitution at *m/z* 858, as indicated by the MS/MS dissociation pattern of the substituted serine octamer. Consistent with this, the MS/MS spectra showed no significant loss of mixed serine/threitol clusters (Figure S10), suggesting that the interactions responsible for stabilizing substituted clusters are substantially weakened in the absence of carboxylate functionality, which may prevent formation of the salt‐bridge network proposed to stabilize the assembly.

Similarly, serine/(S)‐3,4‐dihydroxybutyric acid lithium salt hydrate (4) exhibited only weak substitution, with low‐intensity peaks at *m/z* 856 and 862 corresponding to protonated and lithiated substituted products, respectively (Figure S11). Evaluation of the lithium salt was performed as a control experiment designed to assess the effect of the stock chemical preparation (whether HCl, sodium salt, etc.). These experiments suggest that the observed behavior is independent of the counterion; thus, our results are in fact attributable to the molecular characteristics of the substituents rather than from differences in counterions. Additionally, this indicates that not only the number but also the relative positions of the hydroxyl groups influence substitution efficiency.

Among all compounds examined, GA uniquely displayed substantial substitution accompanied by measurable chiral selectivity. While the present dataset does not establish a strict structural requirement, it suggests that efficient substitution and pronounced chiral discrimination are favored when both a carboxyl group and hydroxyl functionalities are present in a spatial arrangement resembling that of serine, particularly with hydroxyl groups located at the α‐ and β‐positions (Table S1).

This interpretation is consistent with the known zwitterionic structure of serine, which contains COO^−^, NH_3_^+^, and ‐OH groups capable of participating in a combination of ionic interactions, hydrogen bonding, and other noncovalent contacts. Replacement of serine by an α‐hydroxy acid removes the ammonium functionality but retains the carboxylate and hydroxyl groups, resulting in weaker yet still significant interactions when the functional‐group connectivity closely resembles that of serine. Such structural similarity may facilitate incorporation into the Ser_8_ framework and contribute to the enhanced substitution and chiral recognition observed for GA.

## 3 Conclusion

This study examined stereoselective and chiroselective serine octamer formation with α‐hydroxy acids and related molecules. The degree of substitution and stereo/enantio‐specificity depends on the structure of the α‐hydroxy acid group. This was shown with a substrate scope that iteratively varied the number and position of carbons and hydroxyl/carboxylic acid functions. Overall, these reactions emphasize the importance of NH_3_^+^/COO^−^ salt bridges in creating a stable cluster: Replacing serine with an α‐hydroxy acid results in lower stability under CID conditions since these monomers lack amino groups to contribute to the salt bridge. Taken together, our observations support the hypothesis that the octamer's symmetry explains its enantioselectivity.

## Author Contributions

**Keerthana Unni:** writing – original draft, investigation, validation, visualization, formal analysis. **Brison A. Shira:** investigation, conceptualization, methodology, validation, writing – original draft, writing – review and editing. **Dylan T. Holden:** conceptualization, formal analysis, supervision, validation. **Thalappil Pradeep:** supervision, resources, project administration, writing – review and editing, funding acquisition, formal analysis, investigation. **R. Graham Cooks:** conceptualization, funding acquisition, writing – original draft, writing – review and editing, formal analysis, project administration, supervision, resources.

## Supporting information


**Figure S1:** nESI mass spectrum of singly substituted serine octamer [7L‐Ser + D‐TA + H]^+^ in the range of *m/z* 500–1000 in the positive ion mode.
**Figure S2:** n‐ESI mass spectrum of D—tartaric acid showing clusters in the range of *m/z* 500–1000 in the negative ion mode.
**Figure S3:** Tandem mass spectra showing the product of collision‐induced dissociation of single substituted (a) [7L‐Ser + D‐TA + H]^+^, (b) [7L‐Ser + L‐TA + H]^+^, and (c) [7L‐Ser + meso‐TA + H]^+^.
**Figure S4:** Tandem mass spectra showing the product of collision‐induced dissociation of single substituted (a) [7D‐Ser + L‐TA + H]^+^, (b) [7D‐Ser + D‐TA + H]^+^, and (c) [7D‐Ser + meso‐TA + H]^+^.
**Figure S5:** n‐ESI mass spectra of a solution of a mixture of (a) L‐serine and L‐malic acid, (b) L‐serine and D‐malic acid (under the same trapping conditions).
**Figure S6:** n‐ESI mass spectra of a solution containing (a) L‐serine (8 mM) and L‐glyceric acid sodium salt (2 mM), (b) L‐serine (8 mM) and D‐glyceric acid sodium salt (2 mM), (c) D‐serine (8 mM) and D‐glyceric acid sodium salt, and (d) D‐serine (8 mM) and L‐glyceric acid sodium salt. (All these solutions were prepared in the presence of HCl). Compare above to main text Figure 6, which represents the interaction of L‐glyceric acid with L‐serine and D‐serine and a strong chiroselective substitution in the case of glyceric acid. To verify these results, similar studies were required using D‐glyceric acid with L‐serine and D‐serine. However, only the sodium salt form of D‐glyceric acid was commercially available. Therefore, the experiments were repeated using the sodium salts of both L‐glyceric acid and D‐glyceric acid.
**Figure S7:** nESI mass spectra of a solution containing (a) L‐serine (8 mM) and glycolic acid (2 mM), (b) D‐serine (8 mM) and glycolic acid (2 mM).
**Figure S8:** n‐ESI mass spectra of a solution containing (a) L‐serine (8 mM) and L‐2‐hydroxybutyric acid (2 mM), (b) D‐serine (8 mM) and L‐2‐hydroxybutyric acid (2 mM).
**Figure S9:** n‐ESI mass spectra of a solution containing (a) L‐serine (8 mM) and L‐threitol (2 mM), (b) D‐serine (8 mM) and L‐threitol (2 mM).
**Figure S10:** Tandem mass spectrum showing the product of collision‐induced dissociation of single‐substituted [7L‐Ser + L‐threitol + H]^+^.
**Figure S11:** n‐ESI mass spectra of a solution containing (a) L‐serine (8 mM) and (S)‐3,4‐Dihydroxybutyric acid lithium salt hydrate (2 mM), (b) D‐serine (8 mM) and (S)‐3,4‐Dihydroxybutyric acid lithium salt hydrate (2 mM). (Solutions were prepared with HCl). Although (S)‐3,4‐dihydroxybutyric acid lithium salt hydrate contains two hydroxyl groups and one carboxylic acid group, similar to glyceric acid, the different positioning of these functional groups results in only weak substitution, implying that the hydroxyl group must be located at the α‐position relative to the carboxylic acid group for effective substitution.
**Table S1:** Possible interactions of the octamer after replacing the serine with α‐hydroxy acid.

## Data Availability

The data that support the findings of this study are available from the corresponding author upon reasonable request.
