# Supplementary material for: Serine Octamer Substitution Reactions With α‐Hydroxy Acids
Source: Rapid Commun Mass Spectrom. 2026 Jul 2;40(18):e70133. doi: 10.1002/rcm.70133 (PMC13324970; doi:10.1002/rcm.70133)
Supplement: Supplementary file 1 — Figure S1: nESI mass spectrum of singly substituted serine octamer [7L‐Ser + D‐TA + H]+ in the range of m/z 500–1000 in the positive ion mode. Figure S2: n‐ESI mass spectrum of D—tartaric acid showing clusters in the range of m/z 500–1000 in the negative ion mode. Figure S3: Tandem mass spectra showing the product of collision‐induced dissociation of single substituted (a) [7L‐Ser + D‐TA + H]+, (b) [7L‐Ser + L‐TA + H]+, and (c) [7L‐Ser + meso‐TA + H]+. Figure S4: Tandem mass spectra showing the product of collision‐induced dissociation of single substituted (a) [7D‐Ser + L‐TA + H]+, (b) [7D‐Ser + D‐TA + H]+, and (c) [7D‐Ser + meso‐TA + H]+. Figure S5: n‐ESI mass spectra of a solution of a mixture of (a) L‐serine and L‐malic acid, (b) L‐serine and D‐malic acid (under the same trapping conditions). Figure S6: n‐ESI mass spectra of a solution containing (a) L‐serine (8 mM) and L‐glyceric acid sodium salt (2 mM), (b) L‐serine (8 mM) and D‐glyceric acid sodium salt (2 mM), (c) D‐serine (8 mM) and D‐glyceric acid sodium salt, and (d) D‐serine (8 mM) and L‐glyceric acid sodium salt. (All these solutions were prepared in the presence of HCl). Compare above to main text Figure 6, which represents the interaction of L‐glyceric acid with L‐serine and D‐serine and a strong chiroselective substitution in the case of glyceric acid. To verify these results, similar studies were required using D‐glyceric acid with L‐serine and D‐serine. However, only the sodium salt form of D‐glyceric acid was commercially available. Therefore, the experiments were repeated using the sodium salts of both L‐glyceric acid and D‐glyceric acid. Figure S7: nESI mass spectra of a solution containing (a) L‐serine (8 mM) and glycolic acid (2 mM), (b) D‐serine (8 mM) and glycolic acid (2 mM). Figure S8: n‐ESI mass spectra of a solution containing (a) L‐serine (8 mM) and L‐2‐hydroxybutyric acid (2 mM), (b) D‐serine (8 mM) and L‐2‐hydroxybutyric acid (2 mM). Figure S9: n‐ESI mass spectra of a solution contain [file RCM-40-e70133-s001.docx]

**Supporting Information**

**Serine octamer substitution reactions with α-hydroxy acids**

Keerthana Unni ^a, b^, Brison A. Shira ^a^, Dylan T. Holden ^a^, Thalappil Pradeep* ^b^, R. Graham Cooks* ^a^

^a^ Department of Chemistry, Purdue University, West Lafayette, Indiana 47907

^b^ DST unit of Nanoscience (DST UNS) and Thematic unit of Excellence (TUE), Department of Chemistry, Indian Institute of Technology Madras, Chennai 600036, India.

Email: [cooks@purdue.edu](mailto:cooks@purdue.edu), [pradeep@iitm.ac.in](mailto:pradeep@iitm.ac.in)

Table of Contents

| Sl. No | Title | Page no |
| --- | --- | --- |
| Discussion S1 | Materials and Methods | 3 |
| Figure S1 | nESI mass spectrum of [7L-Ser + D-TA + H] ^+^ in the range of *m/z* 500-1000 in the positive ion mode. | 4 |
| Figure S2 | n-ESI mass spectrum of tartaric acid clusters in the negative ion mode | 5 |
| Figure S3 | Tandem mass spectra showing the product of collision-induced dissociation of single substituted of [7L-Ser + D-TA + H] +, [7L-Ser + L-TA + H] +, and [7L-Ser + meso-TA + H] ^+^. | 6 |
| Figure S4 | Tandem mass spectra showing the product of collision-induced dissociation of single substituted [7D-Ser + D-TA + H] ^+^, [7D-Ser + L-TA + H] ^+^, and [7D-Ser + meso-TA + H] ^+^. | 7 |
| Figure S5 | n-ESI mass spectra of L -serine/L-malic acid and L-serine/D-malic acid | 8 |
| Figure S6 | n-ESI mass spectra of L-serine/L-glyceric acid sodium salt, L-serine/D-glyceric acid sodium salt, D-serine/D-glyceric acid sodium salt, D-serine/L-glyceric acid sodium salt. | 9 |
| Figure S7 | n-ESI mass spectra of L-serine/Glycolic acid and D-serine/ Glycolic acid. | 10 |
| Figure S8 | n-ESI mass spectra of L-serine/L-2- hydroxy butyric acid and D-serine/L-2-hydroxy butyric acid. | 11 |
| Figure S9 | n-ESI mass spectra of L-serine/L-threitol and D-serine/L-threitol | 12 |
| Figure S10 | Tandem mass spectrum single-substituted [7L-Ser + L-threitol + H] ^+^. | 13 |
| Figure S11 | n-ESI mass spectra of L-serine/(S)-3,4-Dihydroxybutyric acid lithium salt hydrate and D-serine and (S)-3,4-Dihydroxybutyric acid lithium salt hydrate. | 14 |
| Table S1 | Possible interactions in the serine octamer after substitution | 15 |

1. **Materials and Methods**

All solutions were prepared in LC-MS grade water and loaded into pulled borosilicate capillary nanoelectrospray ionization emitters with an inner diameter of 5 μm. By applying a potential of +2 kV and positioning the nESI 1 cm from the mass spectrometer inlet, droplets were generated and introduced into the LTQ mass spectrometer. Other instrumental parameters included an MS inlet temperature of 159 °C, a capillary voltage of 29 V, and a tube lens voltage of 10 V. Ions were introduced into the trap until the automatic gain target was reached, then held without applying collision energy for 1000 ms prior to mass analysis which was performed using resonance ejection.

Figure S1. nESI mass spectrum of singly substituted serine octamer [7L-Ser + D-TA + H] ^+^ in the range of *m/z* 500-1000 in the positive ion mode.

Figure S2. n-ESI mass spectrum of D - tartaric acid showing clusters in the range of *m/z* 500 – 1000 in the negative ion mode.

Figure S3. Tandem mass spectra showing the product of collision-induced dissociation of single substituted a) [7L-Ser + D-TA + H] ^+^, b) [7L-Ser + L-TA + H] ^+^, and c) [7L-Ser + meso-TA + H] ^+^.

Figure S4. Tandem mass spectra showing the product of collision-induced dissociation of single substituted a) [7D-Ser + L-TA + H] ^+^, b) [7D-Ser + D-TA + H] ^+^, and c) [7D-Ser + meso-TA + H] ^+^.


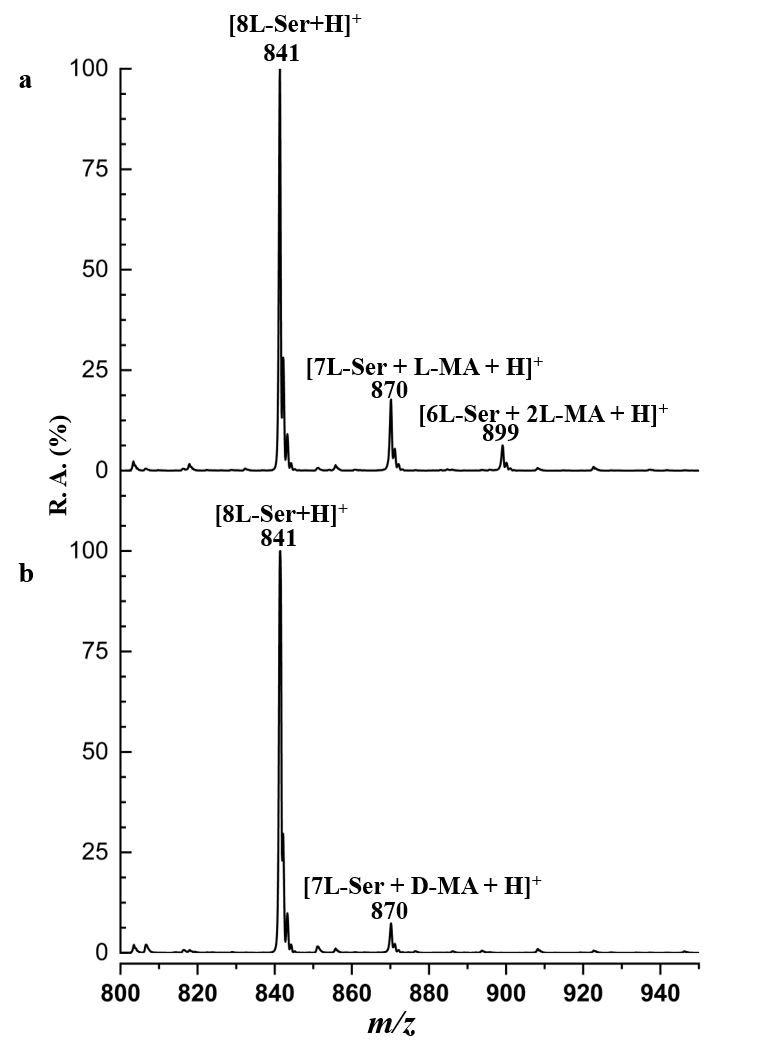


Figure S5. n-ESI mass spectra of a solution of a mixture of a) L-serine and L-malic acid, b) L-serine and D-malic acid (under the same trapping conditions).


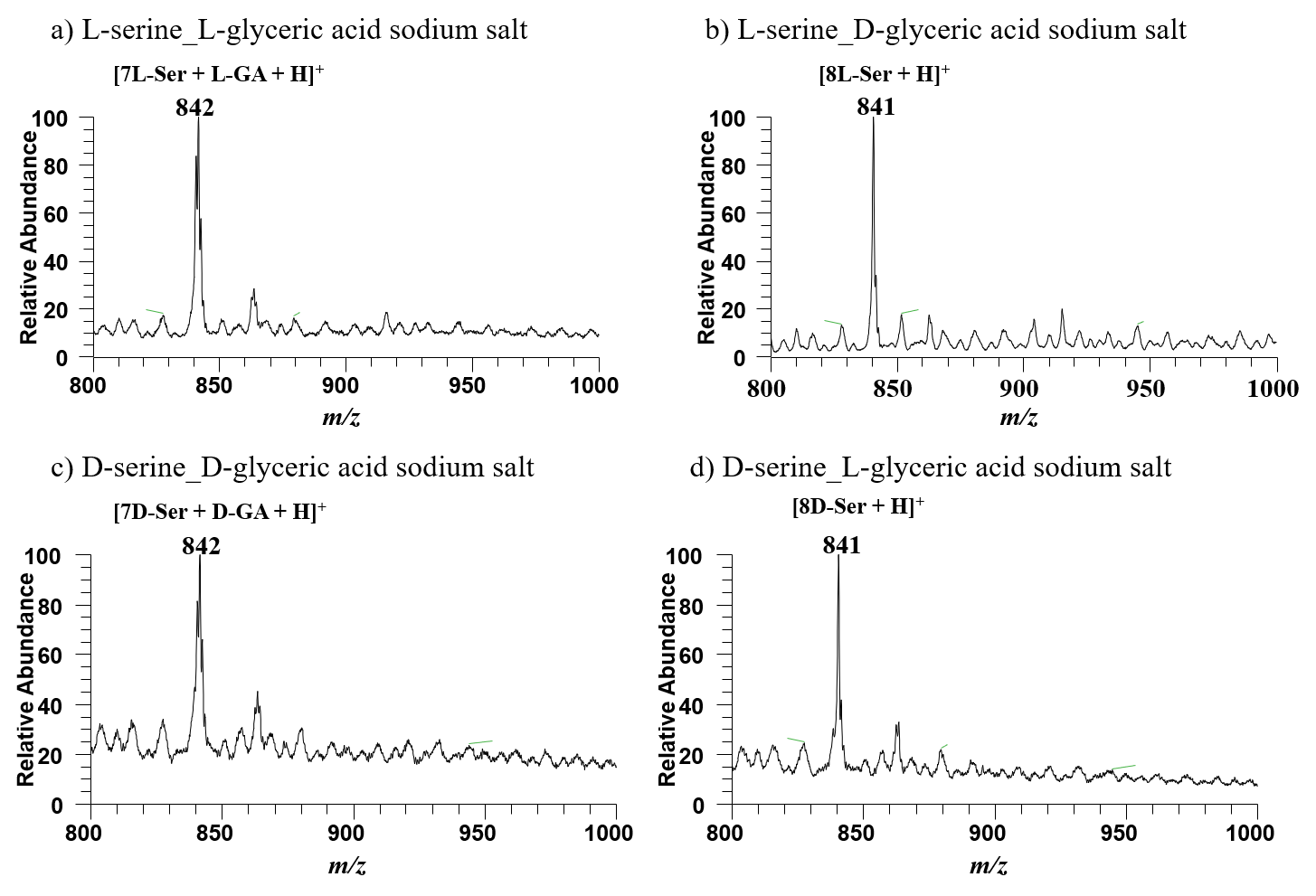


Figure S6. n-ESI mass spectra of a solution containing a) L-serine (8 mM) and L-glyceric acid sodium salt (2mM), b) L-serine (8 mM) and D-glyceric acid sodium salt (2 mM), c) D-serine (8mM) and D-glyceric acid sodium salt, and d) D-serine (8 mM) and L-glyceric acid sodium salt. (All these solutions were prepared in the presence of HCl). Compare above to main text Figure 6, which represents the interaction of L-glyceric acid with L-serine and D-serine and a strong chiroselective substitution in the case of glyceric acid. To verify these results, similar studies were required using D-glyceric acid with L-serine and D-serine. However, only the sodium salt form of D-glyceric acid was commercially available. Therefore, the experiments were repeated using the sodium salts of both L-glyceric acid and D-glyceric acid.


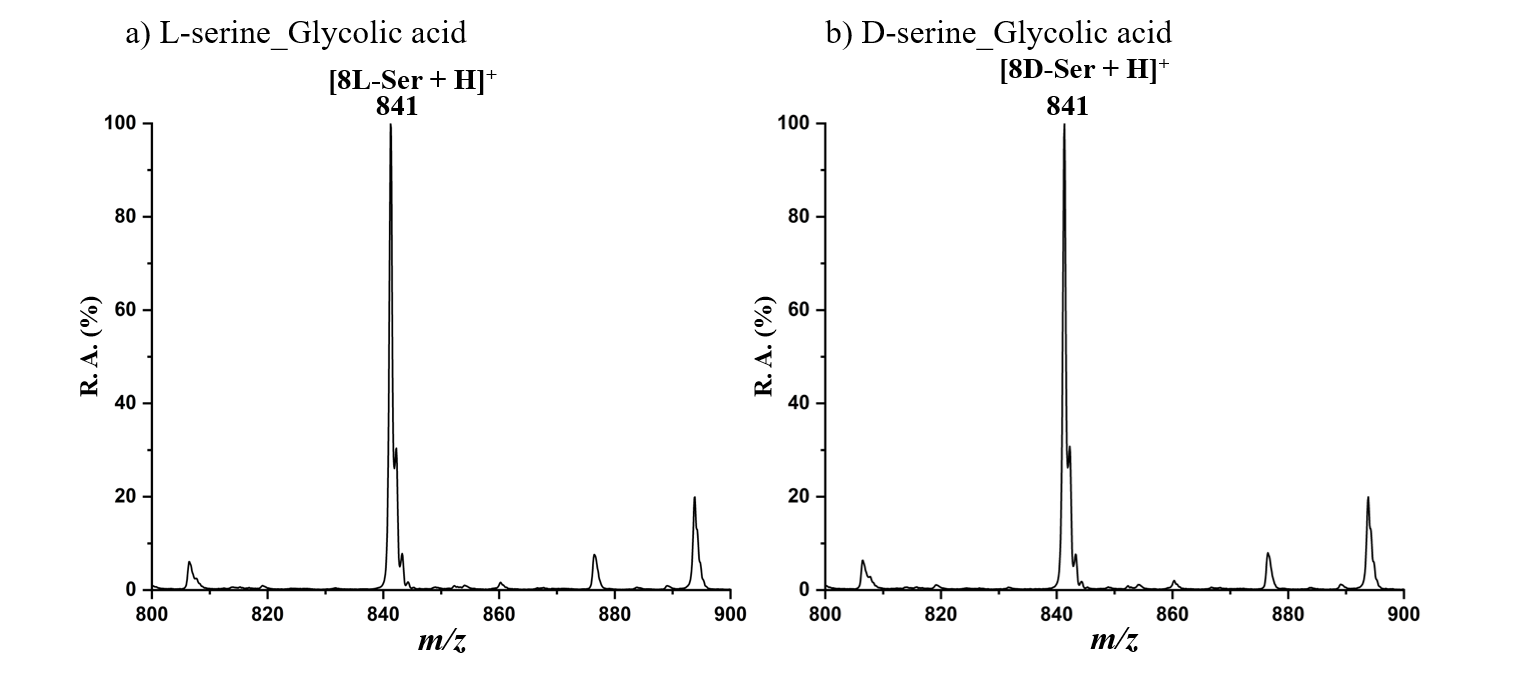
Figure S7. nESI mass spectra of a solution containing a) L-serine (8 mM) and Glycolic acid (2mM), b) D-serine (8 mM) and Glycolic acid (2 mM).


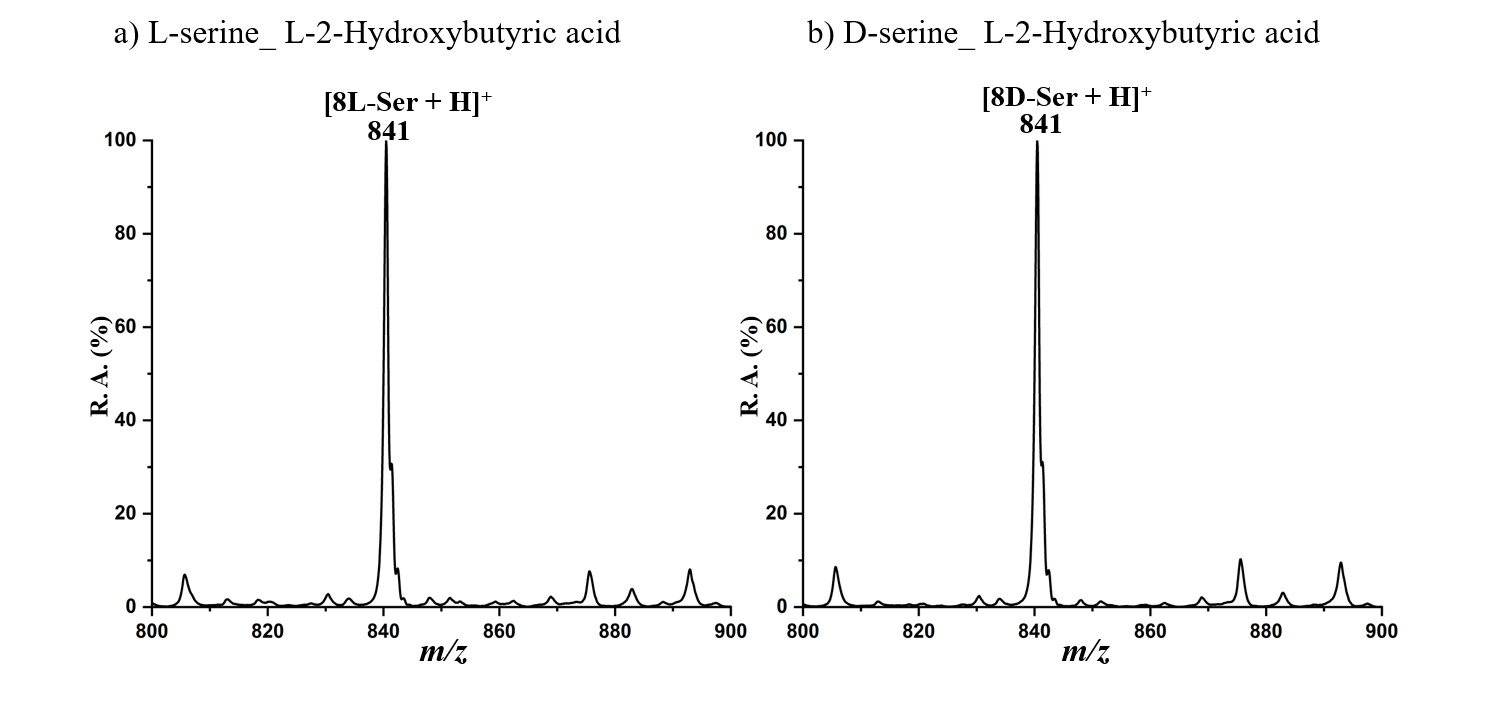


Figure S8. n-ESI mass spectra of a solution containing a) L-serine (8 mM) and L-2- hydroxybutyric acid (2mM), b) D-serine (8 mM) and L-2-hydroxybutyric acid (2 mM).


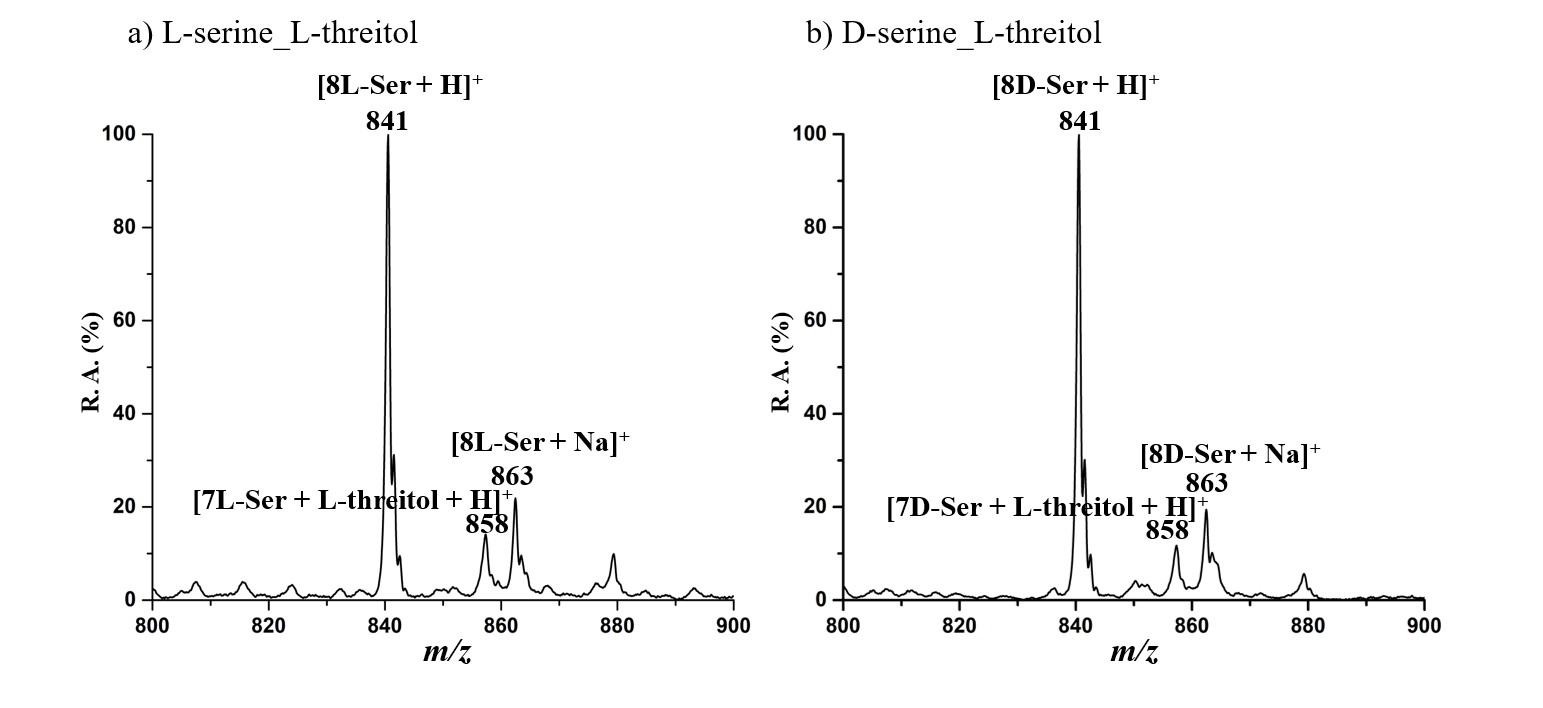
Figure S9. n-ESI mass spectra of a solution containing a) L-serine (8 mM) and L-threitol (2mM), b) D-serine (8 mM) and L-threitol (2 mM).

Figure S10. Tandem mass spectrum showing the product of collision-induced dissociation of single-substituted [7L-Ser + L-threitol + H] ^+^.


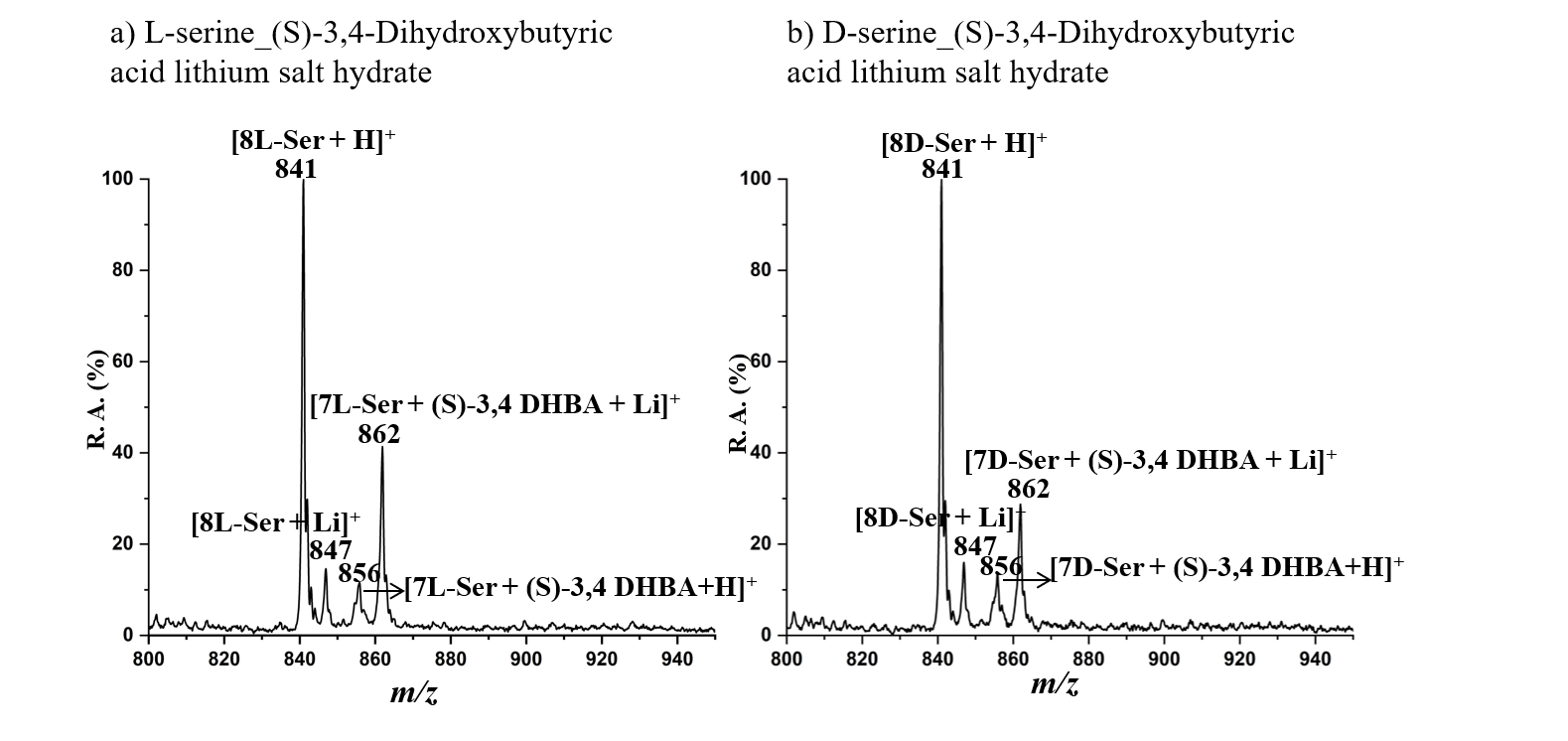


Figure S11. n-ESI mass spectra of a solution containing a) L-serine (8 mM) and (S)-3,4-Dihydroxybutyric acid lithium salt hydrate (2 mM), b) D-serine (8 mM) and (S)-3,4-Dihydroxybutyric acid lithium salt hydrate (2 mM). (Solutions were prepared with HCl). Although (S)-3,4-dihydroxybutyric acid lithium salt hydrate contains two hydroxyl groups and one carboxylic acid group, similar to glyceric acid, the different positioning of these functional groups results in only weak substitution, implying that the hydroxyl group must be located at the α-position relative to the carboxylic acid group for effective substitution.

Table S1: Possible interactions of the octamer after replacing the serine with α-hydroxy acid.

| Sl.no | Molecules | Functional groups | Interactions |
| --- | --- | --- | --- |
| 1 | Ser | -COO^-^ | Ionic bonding (strong) |
| 2 | Ser | -NH_3_^+^ | Ionic bonding (strong), H-bonding |
| 3 | Ser | -OH | Non-ionic bonding (weak), H-bonding |
| 4 | ɑ-HA | -COO^-^ | Ionic bonding (strong) |
| 5 | ɑ-HA | -OH_2_^+^ | Ionic bonding (not as strong as in serine), H-bonding |
| 6 | ɑ-HA | -OH | Non-ionic bond (weak), H-bonding |
| The replacement of serine with ɑ-HA results in weaker binding than serine, but it remains significant. | | | |
